# Agreement between Clinical Assessment and Laboratory Diagnosis of Ringworm in Calves at Auction Markets

**DOI:** 10.3390/ani14030390

**Published:** 2024-01-25

**Authors:** Joachim Spergser, Thiemo Neuhuber, Herfried Haupt, Gerd Kaltenegger, Thomas Wittek

**Affiliations:** 1 Institute of Microbiology, University of Veterinary Medicine, Veterinaerplatz 1, 1210 Vienna, Austria; 2 University Clinic for Ruminants, University of Veterinary Medicine, Veterinaerplatz 1, 1210 Vienna, Austriathomas.wittek@vetmeduni.ac.at (T.W.); 3 Bezirkshauptmannschaft Hartberg-Fürstenfeld, Veterinärreferat, Rochusplatz 2, 8230 Hartberg, Austria; 4 Bezirkshauptmannschaft Leoben, Veterinärreferat, Peter-Tunner-Straße 6, 8700 Leoben, Austria

**Keywords:** ringworm, calves, clinical assessment, laboratory diagnosis, nested PCR, *Trichophyton verrucosum*, MLST

## Abstract

**Simple Summary:**

Cattle ringworm is a mycotic infection of the bovine skin caused by dermatophytes that are also transmissible to humans. To limit the spread of bovine ringworm, calves at auction markets in Styria, Austria, are visually inspected and excluded from auction if they display skin lesions that are typical of ringworm. To investigate whether these clinical assessments correspond to laboratory diagnoses, samples from skin lesions were examined through microscopy, culture, and nested PCR approaches; the relatedness of the isolated dermatophytes was determined using multi-locus sequence typing (MLST). Overall, the clinical assessments were largely supported through the results of the nested PCR laboratory diagnosis, possessing an analytical sensitivity superior to that of the culture approach. Thus, this represents a fast and sensitive diagnostic test for the detection and identification of dermatophytes. Most of the isolated dermatophytes were assigned to a unique *Trichophyton* (*T*.) *verrucosum* MLST genotype, indicating that ringworm in calves at auction was predominantly caused by a single *T. verrucosum* strain.

**Abstract:**

To limit the spread of bovine ringworm, control measures such as movement restrictions are highly recommended. In this context, calves at auction markets in Styria, Austria, displaying skin lesions characteristic for bovine ringworm, are excluded from the auctions. To investigate whether these clinical assessments correspond to laboratory diagnosis, a total of 166 samples taken from skin lesions assigned to the three clinical categories ‘ringworm very likely (v), likely (l) or unlikely (u)’ were mycologically examined using microscopy, culture, and nested PCR followed by amplicon sequencing. Further, the relationships of isolated dermatophytes were determined through multi-locus sequence typing (MLST). Overall, a high agreement between clinical assessment and laboratory results were observed with microscopy and nested PCR, providing more consistent results and molecular detection possessing an analytical sensitivity superior to that of cultural isolation (culture 21.7% vs. nested PCR 48.2%). Phylogenetic analyses revealed that most of the isolated dermatophytes belong to a unique *Trichophyton verrucosum* MLST genotype. In conclusion, clinical assessments were largely confirmed through laboratory diagnosis with nested PCR and sequencing, providing rapid, sensitive, and species-specific detection of dermatophytes in calves at auction markets displaying skin lesions typical for ringworm; this seems to be predominantly caused by a single *Trichophyton verrucosum* strain.

## 1. Introduction

Cattle dermatophytosis, also known as bovine ringworm, is a contagious mycotic infection of the hair and the keratinized layers of the bovine skin, most frequently caused by the dermatophyte *Trichophyton* (*T.*) *verrucosum* [1,2,3,4]. This zoophilic dermatophyte is also transmissible to humans with farmers and their families, farm workers and veterinarians being at risk of infection, sometimes developing severe inflammatory skin lesions [4,5,6]. Bovine ringworm is enzootic in cattle herds in many countries all over the world [4,7,8,9,10,11] and usually occurs in temperate areas during the autumn, winter, and spring months when animals are kept predominantly in-house. Over-crowding, high humidity, low-intensity illumination, and poor stable hygiene promote direct transmission between animals and spreading of the dermatophyte within the herd. Moreover, dermatophytes contaminating the farm environment are a major maintaining factor for cattle dermatophytosis [12].

Clinical symptoms of bovine ringworm vary from mild to severe depending on the virulence of the dermatophyte strain, the host’s immune status, and other factors such as husbandry conditions, management, and production systems [1,2,13]. Ringworm is more prevalent in calves which may be explained with reference to stressors (e.g., weaning, inappropriate diet, parasitic burden, transport, crowding) which are frequently present in this age group [2,9,11]. In general, ringworm is considered a self-limiting disease with symptoms lasting between 4 and 12 weeks. Treatment or vaccination may shorten the duration of clinical symptoms and reduce the risk of spread of infection [12,13,14]. In addition, strict hygienic measures are advised to eliminate dermatophytes from the environment and movement restrictions are recommended to limit the spread of the disease between herds [12].

Conventional methods for the detection and identification of dermatophytes including microscopy and fungal culture are time-consuming, labor-intensive, not always discriminating (microscopy), and require profound expertise in the diagnostician [15]. To circumvent these drawbacks of conventional diagnostics, rapid and sensitive molecular assays such as PCR or real-time PCR are increasingly used for the detection and identification of bovine ringworm dermatophytes in clinical samples [11,13,16,17]. Nevertheless, in most countries, bovine ringworm is considered a relatively harmless disease with little impact on farm economy and animal welfare. Hence, veterinarians usually refrain from performing mycological tests in affected animals and control measures are rarely implemented among veterinary authorities.

In Austria, however, calves gathered for auction in calf markets are visually inspected by official veterinarians at arrival and those displaying skin lesions characteristic for bovine ringworm are excluded from auction. Since this control measure in preventing the spread of bovine ringworm is solely based on clinical assessment, the present study aimed (i) to investigate whether the clinical assessments correspond to laboratory diagnostic results, (ii) to identify the congruence between results of the laboratory methods applied, and further (iii) to gain insight into the epidemiology of bovine ringworm in Austria through determining the phylogenetic relatedness of the dermatophyte strains isolated.

## 2. Materials and Methods

### 2.1. Animals and Sampling Procedures

**Figure 1:**
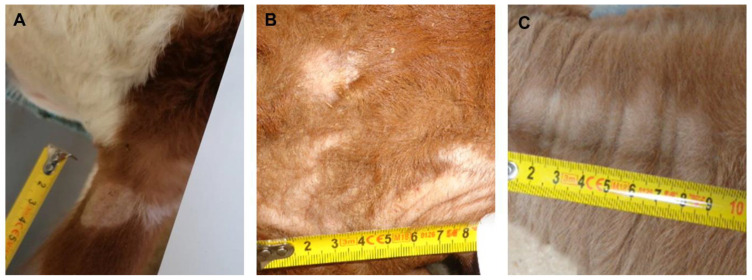
Examples for skin lesions of (**A**) category ‘ringworm very likely’ presenting annular, crusty lesions, and skin thickening on the left ear; (**B**) category ‘ringworm likely’ with non-circular lesions, few crusts and flakes, and very mild thickening of the skin on the calf’s head; (**C**) category ‘ringworm unlikely’ (no skin thickening, flakes or crusts, abrasive hair loss, lateral on the neck).

During the period from March 2020 to March 2021, a total number of 20,259 calves were auctioned at the calf markets of ‘Rinderzucht Steiermark eG’ in Greinbach and Traboch (Styria, Austria). As required, all calves were visually inspected by an official veterinarian on arrival. During these inspections, all calves with superficial skin lesions were assessed, given how likely it is that dermatophytosis (ringworm) is the underlying cause of the skin lesions. Based on the clinical signs, the skin lesions were categorized by the official veterinarian as category v, ‘ringworm very likely‘ (Figure 1A), category l, ‘ringworm likely’ (Figure 1B), and category u, ‘ringworm unlikely’ (Figure 1C). Additionally, the skin areas were photographically documented with a ruler on the picture for later documentation and measurement of the area. After cleaning the skin lesion with 70% ethanol, a superficial skin scraping sample and a hair sample were taken from the margins of the affected area using disposable scalpels. The samples were packed in a paper envelope and sent the same day by messenger service (MedLog^®^, St. Pölten, Austria) to the University of Veterinary Medicine, Vienna, where they arrived the next day for mycological examination. The study has been approved by the institutional animal use and protection committee (ETK-038/02/2020). Since the study did not include invasive or painful procedures, governmental approval was not required. Overall, 166 samples were obtained. The sampled calves were, on average, 95 days old (standard deviation 59.1 days) and mostly Fleckvieh (Simmental) calves (79.5%). Furthermore, crossbred calves (Fleckvieh × Limousin [5.6%], Fleckvieh × Belgian Blue [8.7%]) and calves of other breeds or crossbreeds (6.2%) were recorded. The photographs were assessed using the freeware image measurement software (IC Measure, version 2.0.0.245, https://www.theimagingsource.de/, downloaded on 19 May 2020). The scale was taken from the photographed ruler and the area of the lesion was marked, and from these settings, the software calculated the area of the lesion.

### 2.2. Microscopic Examination and Cultivation

Samples from each individual calf were divided into three portions. The first portion was examined microscopically for fungal structures (e.g., arthroconidia) after incubation in 15% potassium hydroxide for 30 min at room temperature. The second portion was again divided and inoculated onto Sabouraud dextrose agar with chloramphenicol and gentamicin (BBL™, Becton Dickinson, Vienna, Austria) and Dermatophyte test medium agar (BBL™, Becton Dickinson) supplemented with thiamine (4 mg/L) and inositol (100 mg/L) (Sigma Aldrich, Merck KGaA, Vienna, Austria). Both plates were incubated at 30 °C for up to 4 weeks and checked for growth at 4-day intervals. Colonies of dermatophytes were identified according to their macro- and micromorphological characteristics, as previously described [18]. In addition, DNA was extracted from cultured dermatophytes using a GenElute™ Plant Genomic DNA Miniprep Kit, according to the manufacturer’s instruction (Sigma Aldrich, Merck KGaA). For definite species identification of presumptive dermatophyte isolates, amplification and sequencing of the internal transcribed spacer (ITS-1-5.8S-ITS-2 cluster) were carried out as described below (phylogenetic analysis), and the resulting sequences were compared with those available in the NCBI database using the BLAST algorithm (https://blast.ncbi.nlm.nih.gov/Blast.cgi, accessed on 5 December 2023).

### 2.3. DNA Extraction from Samples and Nested PCR

The third portion of each sample was placed into 2 mL tubes incubated overnight at 55 °C in 360 µL ATL tissue lysis buffer and 20 µL proteinase K (both Qiagen). After incubation, a tungsten carbide bead was added to each tube, allowing for sample disruption through high-speed shaking for 2 min using the TissueLyser instrument (Qiagen, Hilden, Germany). Subsequently, DNA was isolated from the sample suspension applying the GenElute™ Plant Genomic DNA Miniprep Kit (Sigma Aldrich, Merck KGaA). For direct molecular detection of dermatophytes in hair and scale samples, a nested PCR was performed as described before [19]. Briefly, a ~900 bp fragment of the ribosomal 18S (3′ end) and 28S (5′ end) genes that included the internal transcribed spacer region (ITS-1, 5.8S, and ITS-2) was first amplified using primers DMTF18SF1 (5′-CCAGGGAGGTTGGAAACGACCG-3′) and DMTF28SR1 (5′-CTACAAATTACAACTCGGACCC-3′). Subsequently, a nested PCR was performed employing primers DMTF18SF1 and DMTFITS1R (5′-CCGGAACCAAGAGATCCGTTGTTG-3′), amplifying the ITS-1 spacer (~400 bp) from the product of the first step—PCR (diluted 1:1 in nuclease-free ddH_2_O). Reaction mixtures for both PCRs contained 12.5 μL (OneTaq^®^ Quick-Load^®^ 2× Master Mix with Standard Buffer (1× containing 25 units/mL OneTaq^®^ DNA Polymerase, 1.8 mM MgCl_2_, and 0.2 mM dNTPs) (New England Biolabs^®^ GmbH, Frankfurt am Main, Germany), 0.5 μL of each primer (20.0 pmol/μL), 9.0 μL ddH_2_O, and 2.5 μL DNA template, yielding a total volume of 25.0 μL. The PCRs were performed in a Mastercycler^®^ nexus PCR thermocycler (Eppendorf Austria GmbH, Vienna, Austria) with cycling conditions, as described in [19]. Nested PCR products were electrophoresed on 1.5% agarose gels, ethidium bromide stained, and photographed using a gel documentation system (Bio-Rad Laboratories GmbH, Vienna, Austria). Amplicons of the nested PCR were then Sanger-sequenced at LGC Genomics Berlin, Germany, for species identification.

### 2.4. Phylogenetic Analysis of Dermatophyte Isolates

The phylogenetic relationship among *T. verrucosum* and *T. mentagrophytes* isolates were determined employing the multi-locus sequence typing (MLST) approach. For this purpose, the ITS rDNA region (ITS1-5.8S-ITS2 cluster), partial *gapdh* gene (glyceraldehyde-3-phosphate dehydrogenase), partial *tubb* gene (β-tubulin), and partial *tef1α* gene (translation elongation factor 1-α) were amplified using primer combinations and PCR protocols, as described previously [20,21]. Reaction volumes of 25 μL were mixed as described for nested PCR. *T. verrucosum* CBS 365.53 (isolated from a cow; country and time of isolation unknown) from which sequences of the MLST scheme were publicly available (Genbank accession numbers: ITS—LR794143; *gapdh*—LR794254; *tubb*—KT155552; *tef1α*—LR792279), and *T. mentagrophytes* GP2015 isolated from a cow with dermatophytosis in 2015 in Austria; these were included in the MLST study as reference strains. Sequences of ITS, *gapdh*, *tubb*, and *tef1α* regions were aligned using ClustalW, trimmed, concatenated (total lengths for *T. mentagrophytes* 2342 bases, and for *T. verrucosum* 2337 bases), and a phylogenetic tree was constructed using the maximum likelihood method and Hasegawa–Kishino–Yano substitution model with bootstrapping (1000 replications) in MegaX [22]. Sequence data were deposited at Genbank and are available under the accession numbers provided in Table S1.

### 2.5. Statistical Analysis

Animal-related data (age, gender, breed) were obtained from the calf market records and transferred into an Excel file. The categorical data from clinical assessment of the lesions and the results of microscopy, culture, and nested PCR were added. For assessing agreement among different diagnostic methods and between laboratory diagnosis and clinical assessment, Cohens Kappa coefficients (κ) and 95% confidence intervals were calculated. Results were interpreted as follows: κ values 0–0.20 as no agreement, 0.21–0.39 as minimal, 0.40–0.59 as weak, 0.60–0.79 as moderate, 0.80–0.90 as strong, and >0.90 as almost perfect agreement [23].

## 3. Results

### 3.1. Clinical Assessment

During the study period, samples were obtained from alopecic skin lesions of 166 calves (total number visually inspected 20,259), resembling a skin lesion frequency of 0.79%. Most of the lesions were found on the head and neck (65.5%) followed by the perianal/tail root area (25.7%). There were no differences between the prevalence of the breeds or gender in relation to the distribution of the 20,259 calves marketed during the period. The official veterinarians scored these 166 lesions into the three categories and collected skin scraping and hair samples: 47 cases were assigned to category v, 55 cases to category l, and 64 cases to category u.

### 3.2. Laboratory Diagnostic Results and Concordance between Laboratory Methods

**Figure 2:**
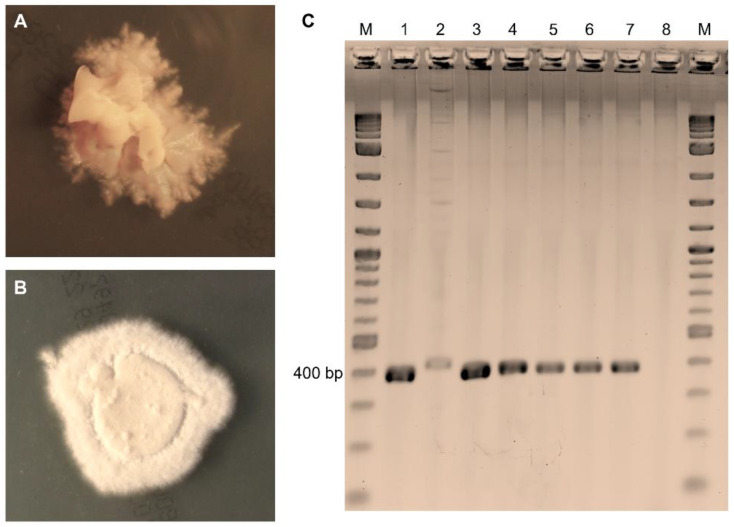
Cultural and molecular detection of dermatophytes in samples taken from calves excluded from auction. (**A**) *T. verrucosum* producing slow growing, cream-colored colonies with a suede-like surface, a raised center, and submerged growth in the periphery after 21 days of incubation on Sabouraud dextrose agar. (**B**) *T. mentagrophytes* presenting with white colonies with a downy or fluffy–powdery surface texture, and brown reverse pigmentation (not shown) after 10 days of incubation on Sabouraud dextrose agar. (**C**) Direct nested PCR on samples producing amplicons at 400 bp for *T. verrucosum* (lanes 1, 3, and 4) and *T. mentagrophytes* (lanes 5–7), and a 420 bp amplicon for *M. canis* (lane 2). Lane M—molecular weight marker; lane 8—negative control.

From a total of 166 samples, 90 (54.2%) were found positive for fungal structures (arthroconidia) through microscopy. Dermatophytes were successfully cultured from 36 (21.7%) samples with 27 (16.3%) being identified as *T. verrucosum*, 8 (4.8%) as *T. mentagrophytes*, and 1 (0.6%) as *Microsporum* (*M.*) *canis*. Nested PCR detected dermatophyte DNA in 80 (48.2%) samples, producing 400 bp amplicons identified through sequencing as *T. verrucosum* (*n* = 59, 35.5%) or *T. mentagrophytes* (*n* = 20, 12.1%), and 1 420 bp amplicon identified as *M. canis* (*n* = 1, 0.6%) (Figure 2). From 28 (63.6%) out of 44 nested-PCR-positive samples that were missed in the culture, non-dermatophyte molds (e.g., *Aspergillus* spp., *Alternaria* sp., *Cladosporium* sp., *Penicillium* sp.) were abundantly isolated. None of the 76 (45.8%) samples found to be negative through microscopic examination were found to be positive in the culture or through nested PCR. The agreement between the microscopy and the culture examinations was moderate (κ: 0.6, CI 0.49–0.71) and the agreement with the PCR was strong (κ: 0.89, CI 0.85–0.93). None of the sample which were diagnosed as negative through nested PCR were found to be positive in the culture, and the agreement between culture and the PCR was weak (κ: 0.45, CI 0.33–0.57). Species identification was identical between the two methods (Table 1). No mixed infections of dermatophyte species were observed, neither through the culture nor through the nested PCR and amplicon sequencing.

### 3.3. Agreement between Laboratory Results and Clinical Assessment

Considering the individual clinical categories, 95.7% of samples formed category v, 72.7% formed category l, but only 7.8% of category u were microscopically positive for fungal elements. Similar results were obtained through nested PCR with 95.7% of samples from category v, 60% of samples from category l, and 3.1% of samples from category u yielding positive results. Of the 36 dermatophyte isolates, a majority (*n* = 33) were recovered from samples of category v and the remaining 3 were cultured from samples of category l (Table 2).

With one exception (category l versus culture—no agreement), the clinical assessment showed moderate, strong, or almost perfect agreement with the laboratory results (Table 3). Taken together, the kappa coefficients for categories v and l (*n* = 102) under microscopy (85 positive samples) is 0.84 (CI 0.76–0.90) (strong agreement); for culture (36 positive samples), this is 0.35 (CI 0.26–0.45) (minimal agreement), and for the nested PCR (78 positive samples), this is 0.89 (CI 0.83–0.95) (strong agreement).

### 3.4. Phylogenetic Relatedness among Dermatophyte Isolates

**Figure 3:**
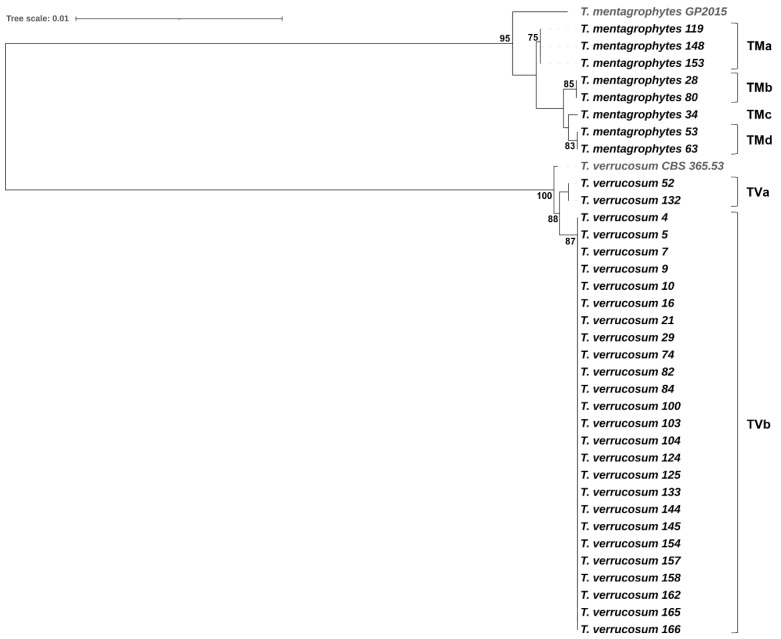
Phylogenetic tree based on concatenated sequences of the ITS rDNA region (ITS1-5.8S-ITS2) and three housekeeping gene fragments (*gapdh*, *tubb*, and *tef1α*) demonstrating the relationships among *T. verrucosum* and *T. mentagrophytes* isolates. *T. verrucosum* CBS 365.53 and *T. mentagrophytes* GP2015 were included as reference strains. The tree was constructed using the maximum likelihood method and the Hasegawa–Kishino–Yano substitution model with 1000 bootstraps (only bootstrap values > 70% are presented). The tree scale indicates the number of nucleotide substitutions per site.

*Trichophyton verrucosum* showed a low level of intraspecific genetic variability. Overall, the 27 *T. verrucosum* isolates were separated into two unique MLST sequence types (TVa, TVb) represented by two substitutions in the ITS rDNA region and a single substitution in the *gapdh* gene fragment. All *T. verrucosum* isolates from the samples of category v (*n* = 25, 92.6%) belonged to MLST genotype TVb, whereas the two *T. verrucosum* isolates from the samples of category l grouped together, forming TVa. In contrast, the eight *T. mentagrophytes* isolates (all from samples of category v) were separated into four MLST genotypes (TMa-TMd); this suggests high intraspecific genetic diversity among *T. mentagrophytes* isolates from cattle with dermatophytosis (Figure 3).

## 4. Discussion

Bovine ringworm is a mycotic skin disease of cattle with a worldwide distribution representing, to a certain extent, a burden on animal and public health, as well as the farm economy. Bovine ringworm is difficult to control when measures such as movement restrictions, hygiene, and treatment of infected animals are not applied; however, vaccination demonstrated high efficacy in the prevention of dermatophytosis in individual animals and at herd level [12,13]. Since the introduction of vaccines against bovine ringworm in the 1970s and their application in many countries worldwide, a reduction in the prevalence of symptomatic ringworm in cattle has been observed [4,24]. The use of vaccines for both prophylactic and therapeutic purposes in Austria may partially explain the low number of confirmed dermatophytosis cases identified in our study (80), resulting in a 0.4% prevalence of symptomatic ringworm in the monitored study population; this included 20,259 calves at auction. However, when considering cases with dermatological lesions (*n* = 166) or clinically suspected dermatophytosis (*n* = 102) only, the prevalence rates of ringworm confirmed using nested PCR were 48.2% or 78.4%, respectively. Similar or higher prevalence rates were reported in Egypt (55.8%) [11], Iran (92.6%) [6], Tuscany/Italy (87.7%) [9], Umbria/Italy (71.7% in cattle aged between 1 and 6 months) [10], and Poland (58.8%) [13]; the latter three studies also included asymptomatic carrier animals. In contrast, a much lower prevalence of 1.6% was observed in Pakistan, likely reflecting the breeding system that is typical for the rural area investigated, with smallholdings of one or two cows of native breeds [25]. 

Although the present study demonstrated high agreement between the clinical assessment of skin lesions categorized as ‘ringworm very likely’ (category v) and laboratory results, a rather moderate agreement was observed between the clinical assessment of skin lesions categorized as ‘ringworm likely’ (category l) and laboratory examination (Table 3); this indicates that clinical cases of category l might require confirmatory laboratory tests if a justification of the exclusion of calves from auction is requested. In this category, microscopy showed a somewhat higher agreement with clinical assessment than nested PCR, which conflicts with previous reports where molecular assays (nested PCR, real-time PCR) showed significantly higher efficiencies in detecting dermatophytes than microscopy [11,13,15]. Another limitation of microscopy is its inability to identify the species of dermatophyte present in the sample, which necessitates additional testing, such as cultural or molecular identification. A high congruency was also noticed between the clinical assessment of skin lesions categorized as ‘ringworm unlikely’ (category u) and laboratory test results, with only 2 out of 64 cases that have been clinically misdiagnosed but carrying *T. verrucosum* being detected through the use of nested PCR. 

Our findings revealed that nested PCR and amplicon sequencing correctly identifies *T. verrucosum*, *T. mentagrophytes*, and *M. canis* in samples that were culture-positive (*n* = 36); this finding agrees with those of other studies, where the nested PCR results were compared with the cultural identification results [11,19]. The present study also proved that nested PCR possesses an analytical sensitivity that is superior to that of cultures, with a 26.5% higher efficiency in detecting dermatophytes present in the samples (culture 21.7% vs. nested PCR 48.2%). Possible explanations for the lower sensitivity of the culture method are the overgrowth of non-dermatophyte molds (observed in 28 out of 44 nested PCR positive samples) known to prevent or obscure the development of slowly growing dermatophytes on agar plates; these lead to not-yet-positive cultures after 4 weeks of incubation, the presence of non-viable dermatophytes in samples from pretreated calves, or the entrapment of dermatophytes in the keratin [11,26].

*Trichophyton verrucosum* was the most frequent dermatophyte detected in our study through the use of nested PCR (*n* = 59), followed by *T. mentagrophytes* (*n* = 20) and *M. canis* (*n* = 1). *T. verrucosum* has been widely described as the predominant etiologic agent of bovine ringworm, with endemic occurrence in many countries worldwide and cattle representing the main reservoir and natural host of this dermatophyte species [4,13,27]. For *T. mentagrophytes* and *M. canis*, on the other hand, wild rodents and dogs and cats in farms have been suggested to be the most likely sources of transmission to cattle [11].

In the present study, an MLST scheme was applied for the intraspecific differentiation of dermatophytes separating 27 *T. verrucosum* isolates into two MLST sequence types, and 8 *T. mentagrophytes* isolates into four MLST sequence types. This sequence-based approach appeared to have a similar or even higher discriminatory power compared to those of PCR fingerprinting methods (PCR-melting profile (PCR-MP) fingerprinting, microsatellite primed PCR (MSP-PCR) fingerprinting) used in previous studies to differentiate *T. verrucosum* or *T. mentragrophytes* isolates [4,28]. The main advantage of MLST in comparison to DNA fingerprinting is, however, the unambiguity and transferability of sequence data, providing comparability between laboratories and with database entries. Altogether, the phylogenetic analyses suggest that ringworm in calves from Styria, Austria, is predominantly caused by clonally related isolates of a single *T. verrucosum* strain circulating in the Styrian cattle population; however, different *T. mentagrophytes* strains may cause dermatophytosis in calves on occasion. 

## 5. Conclusions

In conclusion, the clinical assessments of calves at auction were largely confirmed through laboratory diagnosis with nested PCR followed by amplicon sequencing providing rapid, sensitive, and species-specific detection of dermatophytes in samples from calves displaying skin lesions that are characteristic for ringworm; these seem to be primarily caused by a single *T. verrucosum* strain in calves gathered at Styrian auction markets.

## Figures and Tables

**Table 1:** Laboratory diagnostic results of microscopy, culture, and nested PCR for the detection of dermatophytes in 166 skin samples.

Samples	Microscopy	Culture	Nested PCR	Frequency *n* [%] (Diagnostic Profile)
all categories (*n* = 166)	-	-	-	76 [45.8%]
	+	-	-	10 [6.0%]
	+	-	+(Tv/Tm)	44 (32/12) [26.5 (19.3/7.2)%]
	+	+(Tv/Tm/Mc)	+(Tv/Tm/Mc)	36 (27/8/1) [21.7 (16.3/4.8/0.6)%]
Frequency *n* [%] (diagnostic method)	90 [54.2%]	36 (27/8/1) [21.7 (16.3/4.8/0.6)%]	80 (59/20/1) [48.2 (35.5/12.1/0.6)%]	

Tv—*Trichophyton verrucosum*; Tm—*Trichophyton mentagrophytes*; Mc—*Microsporum canis*.

**Table 2:** Laboratory diagnostic results of microscopy, culture, and nested PCR for the detection of dermatophytes in skin samples of clinical categories v (*n* = 47), l (*n* = 55), and u (*n* = 64).

Clinical Category	Microscopy	Culture	Nested PCR	Frequency *n* [%] (Diagnostic Profile)
v (*n* = 47)	-	-	-	2 [4.3%]
	+	-	+(Tv/Tm)	12 (11/1) [25.5 (23.4/2.1)%]
	+	+(Tv/Tm)	+(Tv/Tm)	33 (25/8) [70.2 (53.2/17.0)%]
Frequency *n* [%] (diagnostic method)	45 [95.7%]	33 (25/8) [70.2 (53.2/17.0)%]	45 (36/9) [95.7 (76.6/19.1)%]	
l (*n* = 55)	-	-	-	15 [27.3%]
	+	-	-	7 [12.7%]
	+	-	+(Tv/Tm)	30 (19/11) [54.6 (34.6/20.0)%]
	+	+(Tv/Mc)	+(Tv/Mc)	3 (2/1) [5.4 (3.6/1.8)%]
Frequency *n* [%] (diagnostic method)	40 [72.7%]	3 (2/1) [5.4 (3.6/1.8)%]	33 (21/11/1) [60.0 (38.2/20.0/1.8)%]	
u (*n* = 64)	-	-	-	59 [92.2%]
	+	-	-	3 [4.7%]
	+	-	+ (Tv)	2 [3.1%]
Frequency *n* [%] (diagnostic method)	5 [7.8%]	0	2 [3.1%]	

v—ringworm very likely; l—ringworm likely; u—ringworm unlikely; Tv—*Trichophyton verrucosum*; Tm—*Trichophyton mentagrophytes*; Mc—*Microsporum canis.*

**Table 3:** Cohens kappa coefficients (κ, 95% confidence interval) characterizing the agreements between the clinical assessment of skin lesion and laboratory methods (microscopy, culture, nested PCR).

Category	Clinical Assessment (*n*)	Positive Microscopy (*n*)	Positive Culture (*n*)	Positive Nested PCR (*n*)
v	47	45 κ: 0.96, 0.90–1.00	33 κ: 0.70, 0.56–0.84	45 κ: 0.96, 0.90–1.00
l	55	40 κ: 0.73, 0.60–0.85	3 κ: 0.06, 0,00–0.12	33 κ: 0.67, 0.61–0.74
u	64	5 κ: 0.92, 0.86–0.99	0 κ: 1.00	2 κ: 0.97, 0.92–1.00

v—ringworm very likely; l—ringworm likely; u—ringworm unlikely.

## Data Availability

The data presented in this study are available on request from the corresponding author. Sequence data were deposited at Genbank and are available under the accession numbers provided in Table S1.

## Supplementary Materials

The following supporting information can be downloaded at: https://www.mdpi.com/article/10.3390/ani14030390/s1, Table S1: Sequence datasets generated and/or analyzed to determine the phylogenetic relatedness of dermatophyte isolates.
