# Supplementary material for: Agreement between Clinical Assessment and Laboratory Diagnosis of Ringworm in Calves at Auction Markets
Source: Animals (Basel). 2024 Jan 25;14(3):390. doi: 10.3390/ani14030390 (PMC10854766; doi:10.3390/ani14030390)
Supplement: Supplementary file 1 [file animals-14-00390-s001.zip › Table S1.pdf]

Table S1: Sequence datasets generated and/or analyzed to determine the phylogenetic relatedness of dermatophyte isolates.

| Reference strain / Genotype | ITS acc. no. | *gapdh* acc. no. | *tubb* acc. no. | *tef1α* acc. no. |
|---|---|---|---|---|
| *T. verrucosum* CBS 365.53 | LR794143 | LR794254 | KT155552 | LR792279 |
| TVa | OR708514 | OR750510 | OR750516 | OR750513 |
| TVb | OR708515 | OR750511 | OR750516 | OR750513 |
| *T. mentagrophytes* GP2015 | OR708516 | OR750512 | OR750517 | OR750514 |
| TMa | OR708518 | OR750512 | OR750519 | OR750515 |
| TMb | OR708518 | OR750512 | OR750518 | OR750515 |
| TMc | OR708517 | OR750512 | OR750518 | OR750515 |
| TMd | OR708517 | OR750512 | OR750517 | OR750515 |
